# When Laughter Hurts and Helps: Associations Between Gelotophobia, Gelotophilia, Katagelasticism and Well-Being

**DOI:** 10.1093/geroni/igaf122.1630

**Published:** 2025-12-31

**Authors:** Robert Reinecke, Diana Mazzarella, Jürgen Maurer

**Affiliations:** University of Lausanne, Neuchâtel, Vaud, Switzerland; University of Neuchâtel, Neuchâtel, Neuchatel, Switzerland; University of Lausanne, Neuchâtel, Vaud, Switzerland

## Abstract

Laughter is commonly seen as a source of positive emotion and a facilitator of social bonding, yet its perception varies based on individual characteristics. Three constructs capture individual differences in how laughter is perceived: gelotophobia (fear of being laughed at), gelotophilia (joy of being laughed at), and katagelasticism (joy in laughing at others). Although these constructs have been predominantly studied in younger adults, their roles in older populations remain underexplored, despite unique social, emotional, and health challenges – including increased social avoidance behavior. This study examined how these constructs relate to well-being – specifically, life satisfaction, social engagement, and mental health – in 1,163 Swiss respondents aged 60 and older from Wave 9 (2021/2022) of the Survey of Health, Ageing and Retirement in Europe (SHARE). The PhoPhiKat-9 questionnaire measured gelotophobia, gelotophilia, and katagelasticism. Life satisfaction, social connectedness, friendship approach and avoidance behaviors, activity frequency, and social support were assessed alongside loneliness (UCLA-3) and depression (EURO-D). Regression models controlling for social, health, and regional factors revealed that higher gelotophobia is linked to lower life satisfaction, greater friendship avoidance, elevated loneliness, and increased depression. Conversely, higher gelotophilia correlates with stronger social connectedness, more frequent approach behaviors, increased activity participation, and a greater likelihood of receiving support. Katagelasticism is associated with reduced social connectedness, lower friendship approach, lower social support, and less frequent social engagement. These findings underscore the significance of laughter-related dispositions for older adults’ well-being and suggest that targeted interventions may enhance social behaviours and mental health in later life.

